# Evaluation of the relationship between non-caries cervical lesions and the tooth and periodontal tissue: An ex-vivo study using micro-computed tomography

**DOI:** 10.1371/journal.pone.0240979

**Published:** 2020-10-28

**Authors:** Go-Eun Lim, Sung-Ae Son, Bock Hur, Jeong-Kil Park

**Affiliations:** Department of Conservative Dentistry, School of Dentistry, Pusan National University, Dental Research Institute, Yangsan, Korea; Danube Private University, AUSTRIA

## Abstract

The purpose of this study was to analyze the relationship between the height and depth of buccal non-carious cervical lesions (NCCLs) and the relationship between the size of buccal NCCLs and clinical crown-root ratio of both buccal and lingual (palatal) sides using micro-computed tomography (micro-CT) images of the teeth and periodontal tissues from the cadavers. The micro-CT images of 56 teeth and their supporting tissues were obtained from 17 cadavers. From these images, the height and depth of NCCLs and the length of the buccal and lingual (palatal) clinical crowns were measured, and the conventional/modified clinical crown-root ratios were calculated. The height and depth ratio of NCCLs were analyzed statistically with the conventional/modified crown-root ratios by Pearson’s correlation and multiple regression. According to the Pearson’s correlation, the height and depth of buccal NCCLs were positively correlated with the modified buccal clinical crown-root ratio (*p* < 0.001 and *p* = 0.013, respectively). The regression model composed of variables of crown-root ratios explained the height of buccal NCCLs, and the prominent factor of the model was the modified buccal clinical crown-root ratio (*p* < 0.001). Moreover, the depth of buccal NCCLs was also explained by the regression model, and its prominent factor was the proportion of modified buccal and lingual (palatal) clinical crowns (*p* = 0.004). The buccal NCCLs were related to the crown-root ratios; particularly, the level of buccal gingival margin could be associated with the formation of buccal NCCLs.

## Introduction

Non-carious cervical lesions (NCCLs) are defined as the loss of hard dental tissue at the cemento-enamel junction commonly located on the buccal and labial tooth surfaces [1,2]. They are revealed as abrasion, abfraction, attrition, and erosion [2]. Attrition is the physiologic wearing of teeth caused by tooth-to-tooth contact; abrasion is the pathologic wear of the tooth through biomechanical frictional processes; erosion is chemically induced loss of tooth by acidic solution; and abfraction is the pathologic loss of tooth because of biomechanical loading forces [3]. It is not easy to diagnose these lesions separately to determine the cause of NCCL, but their appearance and destruction form help to differentiate the related factors [2].

Many studies have suggested the potential causes of NCCLs [2–16]. The formation and/or progression of NCCLs could be caused by a multifactorial etiology [2,11,14]. Some studies have reported that malocclusion could generate an increased stress on the buccal and lingual cervical regions of a tooth than the normal occlusion [4–8]. Other clinical studies have reported an association between the existence of NCCLs and related factors, such as age, gingival recession, occlusion, tooth attrition, and group function [9–14]. A clinical investigation showed that NCCLs appeared most frequently on the maxillary posterior teeth and premolars, and examined the shape and size of NCCLs [13]. Review papers have reported similar conclusions regarding the cause of NCCLs [16,17]. A finite element analysis designed an occlusion model with different alveolar bone levels and showed significantly different stress distribution among the conditions [15]. A prospective clinical study reported that different shapes of NCCLs influenced the progression of the lesion [18].

Abfraction, a manifestation of NCCLs, is caused by dental flexure, occlusal overload, and/or eccentric occlusal forces that exert tensile stress in the cervical region [9]. Therefore, factors influencing dental flexure might be important for the formation and/or progression of abfraction. The tensile/compressive stress generated during tooth flexion on the cervical region of the tooth could be related to the length of the tooth exposed outside the alveolar bone, namely the clinical crown.

The investigations regarding NCCLs have been performed experimentally and clinically at various angles, but there has been no study on the relationship between the actual tooth length and cervical lesions. Therefore, the aim of this study was to determine the relationship between the depth and height of buccal NCCLs and clinical crown ratio of both buccal and lingual (palatal) sides using micro-computed tomography (micro-CT) images of the teeth and periodontal tissues from the cadavers. The null hypothesis was that there was no relationship between the clinical crown ratio and size of NCCLs.

## Materials and methods

The Institutional Review Board of Pusan National University Dental Hospital (IRB, PNUDH-2020-013) approved this study. The micro-CT images of 72 teeth and their supporting tissue or the alveolar bone and gingival tissue were obtained from 17 cadavers donated to the School of Dentistry, Pusan National University. Since all the cadavers were donated for research and education purposes, additional informed consent was not necessary, as confirmed by the ethics committee. The images were taken by a micro-CT (HMXCT 225; X-Tek system, Tring, UK) system at 30 m intervals. The bucco-lingual longitudinal sectional images of the most prominent (mid-) buccal surfaces among the scanned images were selected, including the cross-sectional appearance of the teeth, NCCLs, and supporting tissues. The micro-CT images containing sound teeth without NCCLs or the teeth that lost the periodontal tissue or root apex during tissue preparation were excluded. The total number of teeth included in the study was 56.

**Fig 1:**
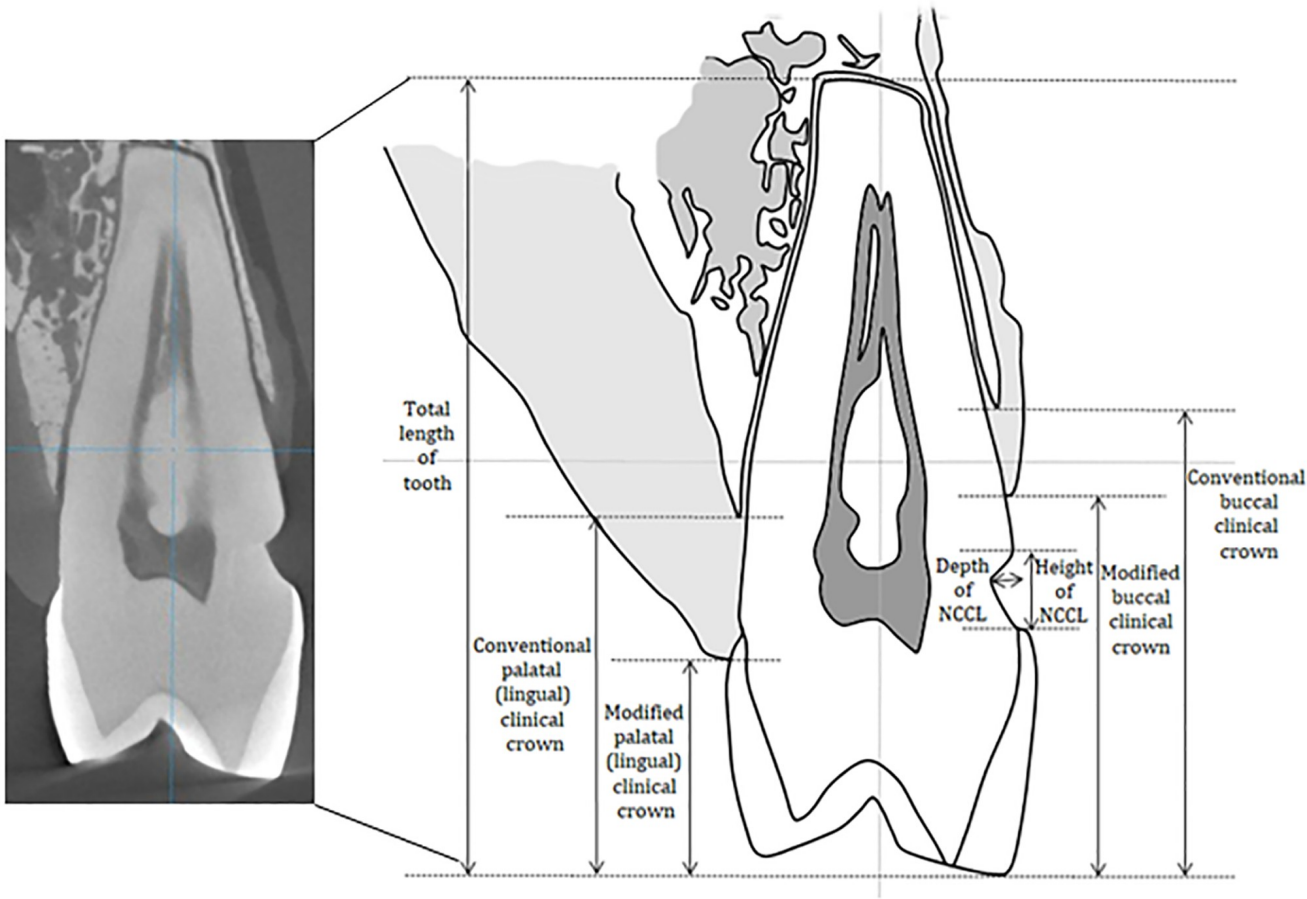
Micro-computed tomography image and its schematic landmarks for measuring length of the clinical crown and non-carious cervical lesion.

Imaging software (OnDemand3D 1.0; Cybermed, Seoul, Korea and ImageJ 1.52a; National Institutes of Health, Bethesda, USA) were used to measure the depth and height of the lesion and length of the teeth from each image. The clinical crown (portion) was defined by the level of supporting alveolar bone [19]. Moreover, a modified clinical crown suggested that the clinical portion was adjusted by the level of gingival margin (Fig 1). The conventional clinical crown-root ratio (C/R ratio) and modified clinical crown-root ratio (mC/R ratio) were calculated from the length of the teeth and the clinical crown. The C/R and mC/R ratios were calculated at both the buccal and lingual (palatal) sides. To compensate for the different sizes of individual samples, the depth and height of the lesion were divided by the total length of teeth to convert them into the following ratios: depth ratio (DR) and height ratio (HR). Furthermore, to determine the effect of different lengths of the clinical crown between the buccal and lingual (palatal) sides, the proportion of buccal and lingual (palatal) clinical crowns (B/L ratio) were calculated. The proportions were also calculated with the conventional clinical crown (B/L ratio) and modified clinical crown (mB/L ratio).

Using the measured and calculated values, statistical analysis was performed by the Pearson’s correlation and multiple linear regression between the DR/HR of NCCLs and buccal/lingual (b-/l-) C/R, b-/l-mC/R, B/L, and mB/L ratios following the enter method. A stepwise method was chosen for the multiple linear regression. The stepwise method deleted the less correlated variables in the regression model so that all variables were not included, unlike the enter method. The Statistic software (Statistical Package for the Social Sciences version 20; SPSS, Chicago, USA) was used for the statistical analysis.

## Results

The micro-CT images of 56 teeth were analyzed. The number of teeth for each position is shown in Table 1. The average size of NCCL is shown in Table 2. The average height of the NCCL was 2.101 mm, and the average depth was 0.734 mm. The C/R ratio was higher than the mC/R ratio.

**Table 1:** Teeth position.

	Maxillary				Mandibular			
	Incisors	Canines	Premolars	Molars	Incisors	Canines	Premolars	Molars
Number of teeth	5	5	15	2	3	5	19	2

**Table 2:** Mean and standard deviation of the height and depth of non-carious cervical lesion and the crown-root ratios.

	Mean	SD
Height of NCCL (mm)	2.052	0.850
Depth of NCCL (mm)	0.701	0.427
Height of NCCL (ratio)	0.114	0.048
Depth of NCCL (ratio)	0.039	0.024
b-mC/R ratio	0.861	0.186
b-C/R ratio	1.408	0.404
l-mC/R ratio	0.601	0.234
l-C/R ratio	1.245	0.583

SD: Standard deviation, NCCL: Non-carious cervical lesion, b-mC/R: Buccal modified crown-root ratio, b-C/R: Buccal crown-root ratio, l-mC/R: Lingual (palatal) modified crown-root ratio, l-C/R: Lingual (palatal) crown-root ratio.

The Pearson’s correlation coefficient between the height and depth of NCCLs was calculated. The coefficient was 0.667. According to this value, the height and depth of NCCLs were positively correlated (p < 0.001), and the coefficient indicated that the height and depth had a moderate relationship.

Table 3 shows the Pearson’s correlation coefficient and significance between the HR/DR ratio and b-/l-C/R, b-/l-mCR, mB/L, and B/L ratios. The HR was positively correlated with the b-mC/R ratio (coefficient 0.586, p < 0.001), b-C/R ratio (coefficient 0.417, p = 0.001), mB/L ratio (coefficient 0.356, p = 0.004), and B/L ratio (coefficient 0.238, p = 0.038). The most correlated ratio with the HR was the b-mC/R ratio. The DR was positively correlated with the b-mC/R ratio (coefficient 0.296, p = 0.013), b-C/R ratio (coefficient 0.266, p = 0.024), and mB/L ratio (coefficient 0.378, p = 0.002). The most correlated ratio with DR was the mB/L ratio.

**Table 3:** Pearson’s correlation coefficient and significance between the height, depth, and other ratios.

Ratio	HR		DR	
	Coefficient	Significance	Coefficient	Significance
b-mC/R ratio	0.586	<0.001	0.296	0.013
b-C/R ratio	0.417	0.001	0.266	0.024
l-mC/R ratio	0.079	0.281	-0.164	0.114
l-C/R ratio	0.14	0.152	0.09	0.255
mB/L ratio	0.356	0.004	0.378	0.002
B/L ratio	0.238	0.038	0.148	0.138

HR: Height ratio, DR: Depth ratio, b-mC/R: Buccal modified crown-root ratio, b-C/R: Buccal crown-root ratio, l-mC/R: Lingual (palatal) modified crown-root ratio, l-C/R: Lingual (palatal) crown-root ratio, mB/L: Ratio between buccal and lingual modified clinical crown, B/L: Ratio between buccal and lingual conventional clinical crown.

The multiple regression summary and its coefficients are shown in Tables 4 and 5, respectively. Each regression model was distinguished by the dependent variables (HR and DR). According to the stepwise method, less correlated variables were deleted from the regression model. The regression model for HR only included the b-mC/R ratio and the other ratios were excluded. The value of R^2^ was 0.344 (R = 0.586). The regression model for DR only included the mB/L ratio and the other ratios were excluded. The value of R^2^ was 0.143 (R = 0.378). The regression models were statistically significant.

**Table 4:** Multiple regression summary between the height ratio/depth ratio and other ratios.

Dependent variables	Included variables	Excluded variables	R	R^2^	Adjusted R^2^	Standard error of estimate
HR	b-mC/R	b-C/R	0.586	0.344	0.332	0.039305
		l-mC/R				
		l-C/R				
		mB/L				
		B/L				
DR	mB/L	b-mC/R	0.378	0.143	0.127	0.02273
		b-C/R				
		l-mC/R				
		l-C/R				
		B/L				

HR: Height ratio, DR: Depth ratio, b-mC/R: Buccal modified crown-root ratio, b-C/R: Buccal crown-root ratio, l-mC/R: Lingual (palatal) modified crown-root ratio, l-C/R: Lingual (palatal) crown-root ratio, mB/L: Ratio between buccal and lingual modified clinical crown, B/L: Ratio between buccal and lingual conventional clinical crown.

**Table 5:** Multiple regression coefficients.

Dependent variables	Included variables	Unstandardized Coefficients		Standardized coefficients	T	Significance
		B (95% CI)	Standard error	Beta		
HR	Constant	-0.016 (-0.066–0.034)	0.025		-0.636	0.527
	b-mC/R ratio	0.151 (0.094–0.208)	0.028	0.586	5.320	<0.001
DR	Constant	0.015 (-0.003–0.032)	0.009		1.661	0.103
	mB/L ratio	0.016 (0.005–0.026)	0.005	0.378	2.997	0.004

CI: Confidence interval, HR: Height ratio, DR: Depth ratio, b-mC/R: Buccal modified crown-root ratio, mB/L: Ratio between buccal and lingual modified clinical crown.

## Discussion

This ex-vivo study examined the relationship between the size of NCCLs and teeth and their periodontal tissues by analyzing micro-CT images of the teeth and periodontal tissues obtained from donated cadavers. Gingival or alveolar bone recession is one of the factors influencing the formation or progression of NCCLs [9–14]. In this study, the effect of gingival recession and exposure to tooth structure on NCCL was investigated by numerical analysis. Since statistical results showed significant correlation between buccal NCCLs and their size (height and depth), the null hypothesis was rejected.

Previous studies were performed by various methods, such as finite element analysis, clinical study, and systematic review, but an ex-vivo study has not been performed before. Measuring images obtained from cadavers have several advantages compared to other studies as follows: high irradiation is allowed to obtain clear images and the radiologic distortion due to head and neck structure might be prevented, thus contributing to accurate measurement and analysis.

Hur et al. [1] reported that NCCLs were located below the cemento-enamel junction. In this context, it is assumed that NCCLs are formed readily on the root dentin than that on the solid enamel structure. Abrasion due to mechanical stimulation or abfraction due to tooth flexure might occur frequently on the root dentin surface. Furthermore, a finite element analysis study reported that lower alveolar bone levels of up to 50% drastically increased stress, and the stress distribution moved toward the apex [15]. The NCCL may be worse at the completely exposed root dentin due to losing the supporting tissues.

In the present study, the alveolar bone loss was replaced by the clinical crown-root ratio. The clinical crown-root ratio can reflect the exposed tooth structure in the oral cavity. The teeth with healthy periodontal tissue have lower C/R ratios, but higher C/R ratios indicate longer clinical crowns and greater exposed root structures. This can be implied as follows: the higher the C/R ratio, the easier it is to be exposed to external stimuli that cause NCCLs; and the higher the C/R ratio, the closer is the center of rotation apically when the teeth are finely bent.

According to the results of Pearson’s correlation, the b-mC/R and b-C/R ratio was more correlated with the HR and DR than the l-mC/R and l-C/R ratio. The correlation between the HR/DR and l-(m)C/R ratio was weak and not significant. The b-(m)C/R ratio was the most statistically significant predictor of the buccal NCCLs. In other words, exposed root dentin is more likely to be affected by NCCLs, and the greater the root is exposed the greater is the probability that the size of NCCL’s height may increase. However, the causality between NCCLs and exposed root dentin is not clear. In the case of maxillary teeth, the buccal alveolar bone is thinner than the palatal alveolar bone, so that gingival recession is more likely to occur at the buccal side. The buccal side of the tooth may be prone to NCCLs. The correlation between the b-(m)C/R ratio and DR was lower than that between HR, but it was statistically significant (p < 0.05). As the depth and height of NCCLs had a moderate correlation, NCCLs could be enlarged in two directions: height and depth.

The correlation between the HR/DR and b-mC/R ratios was greater than that of the b-C/R ratio (Table 3). Mechanical abrasion or erosion only affects exposed dentin beyond the gingival level. When fine flexure of the tooth occurs because of occlusal forces, the fulcrum is located closes to the alveolar bone level. Therefore, mechanical factors may influence the height of NCCL.

To determine the effectiveness of fine rotation of the tooth indirectly, mB/L and B/L ratios were adopted to analyze correlation and linear regression with HR/DR. The (m)B/L ratio can be an indirect indicator of fulcrum or center of rotation. The fulcrum changes when the gingival level shifts to the apex of the root. Moreover, this ratio indicates the difference in gingival recession between buccal and lingual (palatal) clinical crowns. If this ratio is greater than one, the buccal clinical crown is greater than the lingual (palatal) clinical crown. In this study, buccal gingival recession was often greater than the lingual (palatal) gingival recession. The HR and DR were correlated with this value, but revealed weak correlation. The mB/L ratio showed greater correlation than the B/L ratio. It could be affected by the buccal gingival recession mentioned above. Additionally, correlation with this ratio showed similar coefficients for both the HR and DR.

According to the results from multiple regression, the HR and DR showed similar results with the Pearson’s correlation. The HR and b-mC/R ratio showed a moderate relationship through its regression model (R^2^ = 0.344). However, in the case of DR, the mB/L ratio is a prominent factor in the regression model. This suggests that the difference between buccal and lingual (palatal) gingival level affects DR much more than the other ratios. As mentioned above, the change in the fulcrum line may enlarge the DR. However, as the value of R^2^ was low in the DR regression model, a weak relationship was present. A thorough explanation is necessary to investigate the development of depth of NCCL.

Buccal gingival recession and the inclination between buccal and lingual (palatal) alveolar bone levels were prominent factors influencing buccal NCCLs in this study. However, since the regression model only partially explained the phenomenon, other etiology might exist.

It has been shown that abfraction and gingival recession had few relationships with tooth brushing [14,20]. Even though it is not a main factor of NCCLs, it might affect the lesions by auxiliary role [16]. Abusive tooth brushing combined with erosion can cause slight loss of dentin [21]. Moreover, thin buccal alveolar bone could be a predisposing factor of NCCLs on maxillary incisors [22].

The limitation of this study is that the cross-sectional images could not reflect all three-dimensional lesions. The occlusion, diet, habit, brushing, or clinical characteristics of individuals were excluded from the study, so the argument could explain a part of the whole phenomenon. Moreover, since each position of the tooth was neglected, results were put together and were slightly ambiguous. In future research, these limitations should be evaluated in various ways.

## Conclusions

Within the limitations of this study, the level of buccal gingival margin is related to the formation of buccal NCCLs. Because the buccal surface of the tooth seems vulnerable to NCCLs, maintaining healthy periodontal tissue and decreasing the mechanical stimulation to the tooth will be helpful to prevent NCCLs.

## Supporting information

S1 Data (XLSX)
